# Reference-free inferring of transcriptomic events in cancer cells on single-cell data

**DOI:** 10.1186/s12885-024-12331-5

**Published:** 2024-05-20

**Authors:** Batuhan Eralp, Emre Sefer

**Affiliations:** Department of Computer Science, Ozyegin University, Istanbul, Turkey

**Keywords:** Reference-free, k-mer, Differential analysis, Transcriptome, Neoantigens

## Abstract

**Background:**

Cancerous cells’ identity is determined via a mixture of multiple factors such as genomic variations, epigenetics, and the regulatory variations that are involved in transcription. The differences in transcriptome expression as well as abnormal structures in peptides determine phenotypical differences. Thus, bulk RNA-seq and more recent single-cell RNA-seq data (scRNA-seq) are important to identify pathogenic differences. In this case, we rely on k-mer decomposition of sequences to identify pathogenic variations in detail which does not need a reference, so it outperforms more traditional Next-Generation Sequencing (NGS) analysis techniques depending on the alignment of the sequences to a reference.

**Results:**

Via our alignment-free analysis, over esophageal and glioblastoma cancer patients, high-frequency variations over multiple different locations (repeats, intergenic regions, exons, introns) as well as multiple different forms (fusion, polyadenylation, splicing, etc.) could be discovered. Additionally, we have analyzed the importance of less-focused events systematically in a classic transcriptome analysis pipeline where these events are considered as indicators for tumor prognosis, tumor prediction, tumor neoantigen inference, as well as their connection with respect to the immune microenvironment.

**Conclusions:**

Our results suggest that esophageal cancer (ESCA) and glioblastoma processes can be explained via pathogenic microbial RNA, repeated sequences, novel splicing variants, and long intergenic non-coding RNAs (lincRNAs). We expect our application of reference-free process and analysis to be helpful in tumor and normal samples differential scRNA-seq analysis, which in turn offers a more comprehensive scheme for major cancer-associated events.

## Introduction

The detailed analysis of cancer transcriptome has changed our comprehension of tumor dynamics. Such analysis is currently being utilized in studying tumor progression dynamics and their diagnosis, mainly because of the broader and cost-effective appearance of next-generation sequencing (NGS) techniques. The earlier transcriptomic analysis mainly utilizes DNA microarrays while focusing on genes encoding for protein. Newer bulk RNA-seq and single-cell RNA-seq (scRNA-seq) datasets provide us with a more comprehensive analysis and breakdown of gene expression. Even though RNA-seq and scRNA-seq can identify a higher number of transcripts than the typical DNA microarrays, their focus in cancer cells has again been over annotated genes. However, such analysis over annotated genes may not include a significant number of unannotated endogenous reverse transcription elements, non-coding RNAs, mRNA isoforms, as well as unannotated transcripts of bacteria and viruses [1]. In this case, classical RNA-seq analysis techniques ignore a significant degree of knowledge of those transcripts. More recently, such ignorance has started to be taken more seriously by researchers. Recent studies have focused on inferring the cancer dynamics and mechanisms via quantitatively analyzing the transcripts. As a result, these studies have identified a great many numbers of cancer-associated transcripts which include splicing variants [2], viral RNA [3], bacterial RNA [4], small nucleolar RNAs (snoRNAs) [5], and long intergenic non-coding RNAs (lincRNAs) [6].

The remaining missing RNA diversity is also due to genomic mutations and duplications of the blacklisted regions, since those regions may not be inferred via traditional techniques [7]. To our best knowledge, none of the existing approaches can simultaneously analyze and infer this whole set of different types of mutational knowledge from RNA-seq or scRNA-seq transcriptomic datasets. Such a missing analysis is mainly due to more classical transcriptome analysis requiring a reference genome, which compares sequence datasets to reference sequences. Correspondingly, we lose a significant amount of unannotated genetic knowledge which could not be assessed via the comparison tools. In cancer cells, each tumor cell has almost a distinct transcriptome differing from the non-cancer tissues transcriptome in a number of means. Therefore, techniques which are not based on reference sequence alignment can be important and remarkably beneficial.

Here, we have applied a recently-proposed exhaustive technique DE-kupl [8] that carries out differential analysis of scRNA-seq transcriptome datasets via smaller k-mers. DE-kupl does not use reference sequences and does not depend on aligning sequences. So, it may identify novel RNA and RNA isomers occurring within the datasets at a nucleotide resolution. This is especially functional and important for transcripts which cannot be easily aligned by more classical techniques, namely chimeric RNA and RNA from repeated sequences. We have compared the whole set of non-reference events identified in esophageal cancer (ESCA) and glioblastoma tissues with the normal tissues that are located near cancerous tissues by DE-kupl. Our results do not significantly change if we use different tools such as MINTIE [9] and TAP [10] instead of DE-Kupl. Those original transcriptome events in cancer cells are mainly due to mutations in non-coding and coding regions. As a result, we have also identified antigens specific to tumors that have therapeutic potential. Additionally, those novel events have been shown to be critical for diagnosing tumors, the prognosis of tumors, and the infiltration of the immune system.

### Related work

The existing strategies for bulk RNA-seq and single-cell RNA-seq analysis do not completely consider an extensive set of transcript diversity. A commonly-used technique aligns or pseudo-aligns RNA-seq reads over a reference transcriptome in quantifying transcripts [11]. Even though those techniques could be utilized to detect isoform-switching events, such analysis is restricted to transcripts occurring over the input reference [12]. A different method tries to build full-length transcripts, either de novo or reference-based. Even though those procedures could infer the previously undiscovered transcripts, they cannot fully analyze the true diversity of transcription since small-scale variations are ignored by them, such as SNPs, indels, and edited bases, as well as they have a difficulty in handling repeat-including transcripts. Another set of procedures focuses on discovering specific events, such as allele-specific expression, circular RNAs, fusion transcripts, intron retention events, alternative polyadenylation events, or splicing events. Some examples of these variation detection techniques are CICERO [13], MINTIE [9], TAP [10], DE-kupl [8], etc. Strategies that combine a diverse set of analysis tools for a comprehensive analysis of transcriptome cannot be easily implemented and they are not fully exhaustive [14]. Among the existing work on cancer transcriptome analysis, [15] focuses on discovering transcriptomic events only in esophageal cancer’s bulk RNA dataset. Their study is limited to applying De-Kupl for differential RNA analysis.

## Materials and methods

### Datasets

Discovery Datasets: We have obtained 64 ESCA single-cell RNA-seq samples from [16] which includes 60 esophageal cancer tissues and 4 neighboring normal tissues, over 60 individuals. When needed, we have converted files in bam formats to fastq file formats via Picard tools [17]. CutAdapt software [18] has been used to trim sequences with low quality and adapter sequences. By applying a similar procedure, we have also processed brain cancer (glioblastoma) single-cell RNA-seq samples over 25 cancer tissue samples and 5 neighboring normal tissue samples as a discovery dataset [19].

Validation Datasets: We have obtained the validation dataset for ESCA with accession PRJNA374673 from SRA database [20]. This validation dataset includes 40 ESCA cancer tissues and 40 matching non-cancer tissues. We have obtained the fastq files from SRA via SRAtoolkit software (https://hpc.nih.gov/apps/sratoolkit.html). CutAdapt software has again been used to trim sequences with low quality and adapter sequences. By applying a similar procedure, we have also obtained brain cancer samples from SRA database with accession PRJNA869596, over 20 cancer tissues and 20 normal tissues as a validation dataset.

### DE-kupl software pipeline

As a first filter, k-mers appearing fewer than 5 times as well as occurring in less than 10 samples have been removed to significantly lessen the effect of sequencing errors, while keeping almost all of the esophageal cancer-associated mutations. In the second filter, the whole set of k-mers occurring in the reference genome has been removed. No variation is contained in those k-mers since those k-mers are the same as the reference sequence. By utilizing the filtering approach, we are able to concentrate on novel transcripts without any annotation or transcripts with mutations. Afterwards, k-mer counts are normalized. In this case, the normalization factor of k-mer count is computed by the median of the sample count to the pseudo-reference count, that results from the calculation of the geometric mean of each k-mer across the whole set of samples. Rectification and normalization have been used to remove the unequal structure of the dataset generated by the discrepancy among samples.

While carrying out differential expression (DE) analysis, we have identified k-mers which are remarkably differentially expressed between normal and ESCA tissues by using Limma Voom [21] algorithm. Similar differential expression analysis has also been applied to glioblastoma. After applying multiple test corrections, we have chosen statistically significant k-mers with a *p*-value lower than 0.05 and log2FC value greater than 1 as differentially-expressed k-mers. In this analysis, we have combined the identified k-mers with statistically significantly different expression values into longer sequences called contigs. In addition to k-mer analysis, we have also carried out a quantitative analysis more conventionally and directly over gene levels. We have utilized Kallisto software [22] and Gencode v34 transcripts while measuring the gene level expressions, and incorporated transcripts TPM (Transcript Per Million) values from the identical gene. We have again carried out differential expression analysis by using Limma and following the procedure described above.

### Annotation of contigs

DE-kupl does not depend on reference sequences, which is one of its main premises. Differentiation among contigs containing different types of variations and the contigs proposing novel transcripts can be mainly achieved by using sequence alignment annotation analysis on the whole set of contigs sequences. We match contigs to the human genome for the contigs sequence assembled via DE-kupl program, by GSNAP (Genomic Short-read Nucleotide Alignment Program) which is a tool to align single-end and paired-end reads to a reference genome. Moreover, the exact genomic position, adjacent genes, Differential Usage (DU) status [23], and functional intervals such as introns, exons, or intergenic regions have also been provided [23]. Repetitive sequences are always sensitive and difficult for each existing aligner due to them aligning to more than one genome position. To handle those repeated sequences, BLAST method is used for aligning them to the DFAM database [24]. Lastly, contigs are classified into event classes such as lincRNA, splitting, duplication, polyA, introns, SNV (Single nucleotide variant), splicing, and unmapped. We have not called any anti-sense events as our datasets are not stranded.

### Event categories-based clustering of samples

The features of distinct transcription categories in ESCA tissues have been analyzed by a number of clustering techniques. Firstly, PyComplexHeatmap Python package is used to analyze the dissimilar transcription categories expression distributions over ESCA and normal tissues as well as over glioblastoma and normal tissues [25] Non-negative Matrix Factorization (NMF) approach [26] has been used to group tissue samples and examine them, which resulted in the analysis of variations in non-coding and coding intervals. In this case, intuitive evaluation can be achieved by deciding whether a non-coding and coding variation in ESCA and glioblastoma tissues shows different subtypes by NMF clustering.

### Gene Ontology-based functional enrichment in terms of host genes and event categories

In transcriptome analysis literature, differentially-expressed genes are commonly used as part of enrichment analysis of biological functions, in turn which is mainly to detect atypical biological functions as part of illness prognosis. Nonetheless, a small number of research analyze biological functions at transcript level. Therefore, similar to the traditional analysis method, differentially-expressed genes are used for enrichment of biological functions as a control. In our case, we carry out the significant and crucial transcriptome events functional analysis. We use clusterProfiler R package [27] and GSEApy package [28] for gene ontology [29] functional enrichment by determining the corresponding host gene for every transcriptome event, where the significance level is determined by an adjusted *p*-value $< 0.05$ (Bonferroni corrected).

### Relationships between variants and host genes in terms of expressions

Differentially-expressed transcriptome events are not consistent with the host genes dynamics all the time. For instance, in some cases, we observe strong expression of transcriptome events whereas host genes are not expressed as strongly. A codirectional relationship is formed between the consistently-behaving transcription events and their corresponding host genes, which implies that differentially-expressed transcripts constitute the main cause of diverse host gene expression. We establish Differential Usage (DU) pairs over transcripts that exhibit behaviours different than their host genes. Such different transcripts might be considered to differentiate from their host genes or the remaining transcript parts, which indicate biological functional problems and specific biological variations. In this case, we have analyzed those 2 categories of transcript-host gene relationship pairs one at a time.

### Survival analysis of event categories

We have obtained the clinical knowledge by using GDC portal (https://portal.gdc.cancer.gov/projects/TCGA-ESCA), which includes status as well as final survival duration. Afterwards, univariate Cox regression [30] and multivariate Cox regression [31] have been applied over every event category for evaluating the differential events prognosis values. We have carried out survival analysis by utilizing lifelines Python package [32]. For every contig, we calculate *p*-values and hazard ratios (HR). After such calculation, contigs with *p*-value $< 0.05$ and HR $> 1$ are treated as the prospective risk components. Lasso Cox regression has been originally run with glmnet Python package [33] to select contigs for multivariate Cox regression, by applying it to every contig category in an independent manner. Following such an independent application, we have established multivariate model by utilizing the chosen contigs. We divide the patients into low and high-risk classes over all risk scores median values to represent in Kaplan-Meier (KM) curves [34].

### Detection of neoantigens

Antigens specific to tumors can be considered as abnormal polypeptides which can solely be observed on tumor cell surfaces [35]. These polypeptides are immunogenic, suggesting that they might be identified and presented via immune cells prior to killing tumor tissues. A new protein that forms on cancer cells when certain mutations occur in tumor DNA is called a neoantigen. While discovering prospective antigens having mutations within the DNA’s coding region which have specifically been expressed in tumor tissues, conventional techniques mainly integrate transcriptome sequencing with whole exome sequencing. Besides, non-coding areas of DNA may also generate transcripts and be translated into peptides. Moreover, a number of new genes which can potentially generate antigens specific to tumors have not been discovered yet. Correspondingly, we have analyzed the transcription events specific to tumors across coding and non-coding regions in detail. Such analysis and evaluation procedures are composed of 2 parts. Initially, our results over cancer tissues are compared with normal tissues and search for antigens which are uniquely identified over ESCA and glioblastoma tissues. Secondly, we have assessed the affinities of all peptides by using netMHCpan version 4.0 [36] and have identified antigens specific to tumors with high binding affinities for MHC-I molecules.

### Cohort validation

We have independently verified the forecasted tumor-specific antigens to guarantee our screened tumor-specific antigens are reproducible. We have extracted the contig sequences by applying DE-kupl with the identical independent process over the discovery dataset. Afterwards, we use Pairwise2 from the Biopython package [37] to carry out a pairwise sequence alignment for the contigs over the validation dataset, independently for each tumor-specific antigen inferred over ESCA cohort. When there are multiple alignment sequences corresponding to a tumor-specific antigen, we select the antigen whose alignment score is the greatest. Then, we focus on analyzing whether the expression values between normal tissues and ESCA tissues differ in the validation dataset for the matching contig sequences.

### Sequence alignment views

For each cohort, we have generated meta-bam alignment files for normal and tumor tissues to better visualize the events. To achieve this visualization goal, 1 million reads have been randomly sampled over each subcohort’s fastq file and the alignment of accumulated reads to genome (GRCh38) is carried out by running STAR RNA-Seq aligner [38] with its default parameters. Afterwards, Integrative Genomics Viewer (IGV) is used to visualize BAM files [39].

## Results

### Differential analysis of genes and contigs

We have examined events which are expressed differentially across cancer and normal tissues at contig and gene levels. Comparison of differential expression events across various dimensions summarizes the similarities and disparities among conventional gene level and accurate base level analysis. We stick to the preprocessing steps of the well-studied approaches in terms of gene level analysis. Such steps incorporate transcript quantification, transcript unification from the identical gene, and finally raw count estimation at a gene level. We normalize gene level expression profiles to less the library sequencing and gene length effects, where TPM values are generated by raw counts. Afterwards, DE-kupl approach is utilized in contig level analysis. DE-kupl has an in-house mechanism for standardization which uses k-mer counts to fix the contig quantification. Lastly, Limma Voom approach is used to extract genes and differentially-expressed contigs.

**Fig. 1:**
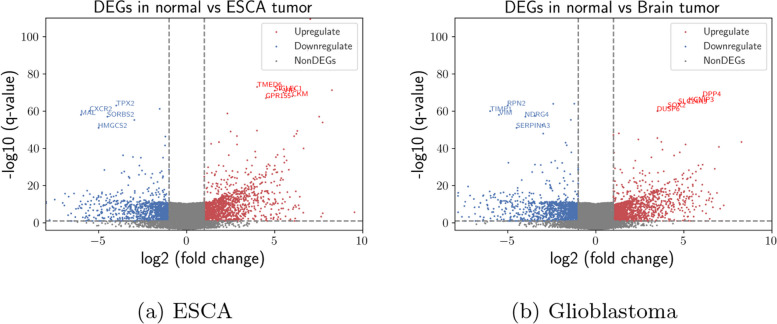
Gene vs contig level analysis. Differentially-expressed genes distribution between normal and tumor cells in ESCA and glioblastoma. Blue and red colors show downregulated and upregulated genes respectively

As seen in Fig. 1a for ESCA tumors, 1623 upregulated genes and 1424 downregulated genes have been screened over a total of 23213 genes. 51732541 differentially-expressed k-mers have been identified at the contig level. We have assembled those k-mers into 432651 differentially-expressed contigs in ESCA tissues where 262131 upregulated contigs and 170520 downregulated contigs exist. Then, genes are related to differentially-expressed contigs. The inferred downregulated and upregulated contigs are related to 10131 and 6501 genes, respectively. Similarly, Fig. 1b plots the distribution of upregulated and downregulated genes for glioblastoma. In ESCA data, we found TMED6, GPR155, SIGLEC1, VIP, and CKM to be differentially the most upregulated, which have previously been found to be effective in ESCA formation and prognosis [40]. We found TPX2, SORBS2, HMGCS2, CXCR2, and MAL to be differentially the most downregulated genes. Among them, TPX2 depletion is a well-known biomarker in ESCA cells, leading to reduced cancer cell growth and invasion ability [41]. CXCR2 has also been previously found to mediate the angiogenic effects in intestinal microvascular endothelial cell [42]. While considering glioblastoma, we found SOX2, DUSP6, SLC24A3, KCNIP3, and DPP4 to be differentially the most upregulated, which have previously been found to be effective in glioblastoma formation and prognosis. Among those genes, SOX2 is a well-established stem cell transcription factor needed to induce and maintain stemness properties of glioblastoma cancer cells [43]. DUSP6 is also known to be actively involved in oncogenesis showing unexpected tumor-promoting properties in human glioblastoma, contributing to the development and expression of the full malignant and invasive phenotype [44, 45]. In terms of downregulated genes in glioblastoma, we found NDRG4, SERPINA3, RPN2, VIM, and TIMP1 to be differentially the most downregulated genes [46]. For instance, expression change in RPN2 is known to be effective in multiple cancer type formation and poor outcome [47].

**Fig. 2:**
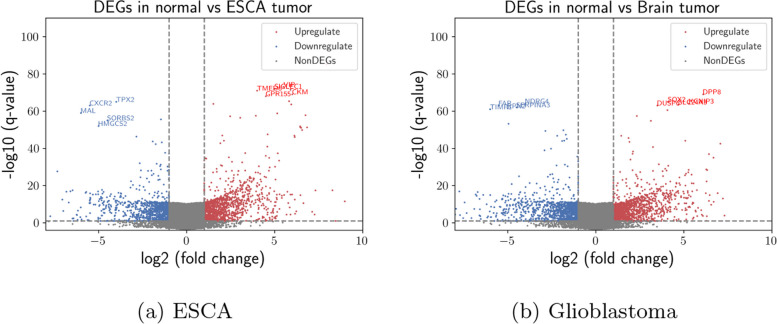
Gene vs contig level analysis over validation dataset. Differentially-expressed genes distribution between normal and tumor cells in ESCA and glioblastoma. Blue and red colors show downregulated and upregulated genes respectively

When we repeat analysis in Fig. 2a, b over validation dataset, we obtain almost same results where we again observe gene overexpression as seen in Figure. One thousand five hundred seventy-seven upregulated genes and 1384 downregulated genes have been screened over all genes. Most upregulated and downregulated genes for both ESCA and glioblastoma are almost same between discovery and validation datasets. Over the validation dataset, 49824871 differentially-expressed k-mers have been identified at the contig level. We have assembled those k-mers into 422133 differentially-expressed contigs in ESCA tissues where 263354 upregulated contigs and 158779 downregulated contigs exist. Then, genes are related to differentially-expressed contigs. The inferred downregulated and upregulated contigs are related to 10215 and 6553 genes, respectively. Similarly, Fig. 2b plots the distribution of upregulated and downregulated genes for glioblastoma. The difference between discovery and validation datasets is not statistically significant for both ESCA and glioblastoma according to Wilcoxon test [48].

**Fig. 3:**
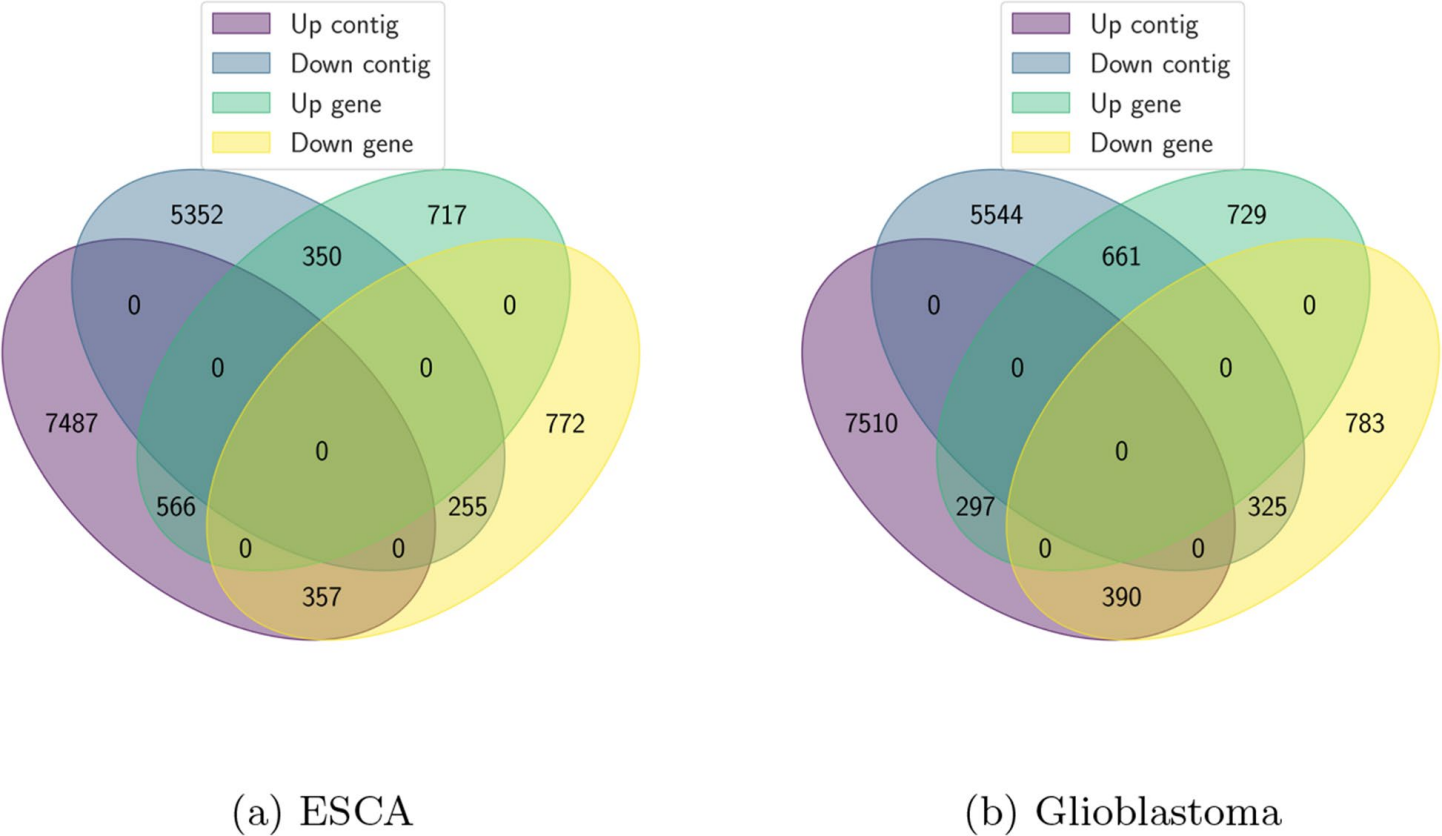
Contig level vs gene level analysis. Differentially-expressed genes and contigs overlap and relative comparison in ESCA and glioblastoma

As seen in Fig. 3a, those genes that are mapped at the contig level incorporate a greater knowledge than the genes inferred via more traditional gene level studies in ESCA. The differentially-expressed genes have disclosed consistent results, where 566 ($34.87\%$) upregulated genes as well as 255 ($17.90\%$) downregulated genes are identified across these two different analysis methods. On the contrary, the conventional gene level analysis approach cannot detect host genes over significantly different contigs, so they should be inferred at the contig level. In this case, we have identified 5352 downregulated and 7487 upregulated contigs over host genes. Conventional gene level analysis could not uncover those genes since different expression is observed solely on a few transcripts. We have also inferred differential usage cases. For instance, 357 differentially-expressed genes have been downregulated but also generated upregulated contigs. Even though those host genes and contigs exhibit distinct expression patterns and are significantly related to ESCA, they cannot be accessed via conventional gene level research. Similar contig level vs gene level analysis for glioblastoma is shown in Fig. 3b.

### Classification of transcriptome events

We have identified 431251 contigs via DE-kupl technique. Those identified contigs are derived from different genome regions corresponding to distinct transcriptome events. We have completed the set of annotations by mapping those contigs to the human genome and then classifying them into multiple distinct transcriptome events depending on the type of variation and genome position. In this case, conditions for such classification are shown in Table 1 for both ESCA and glioblastoma. To test the robustness of our analysis on transcriptome events, we also run our analysis by using MINTIE [9] and TAP [10] as well, where conditions for such classification are shown in Table 2 for both ESCA and glioblastoma. In our results, we focus all our analysis on DE-Kupl since results from other techniques are similar.

**Table 1:** Events classification/categorization conditions for both ESCA and glioblastoma by DE-Kupl

		Event count	
Event class	Condition for inclusion	ESCA	Glioblastoma
Repeats	Tandem repeats or multiple hits	69	91
LincRNAs	Positioned in intergenic regions	137	173
Introns	Positioned in intronic regions	522	532
Splices	Spliced	588	593
Polyas	Unmapped PolyT head or polyA tail	31	38
Split	Partially-mapped or chimeric	5	6
Unmapped	Unmapped	72	67
Snvs	Mapped, contains SNV	177	175
Neos	Tumor-specific (expression=0 in normal)	351	361

**Table 2:** Events classification/categorization conditions for both ESCA and glioblastoma by MINTIE and TAP

	MINTIE		TAP	
Event class	ESCA	Glioblastoma	ESCA	Glioblastoma
Repeats	65	93	60	94
LincRNAs	130	175	135	170
Introns	513	550	515	536
Splices	577	584	588	591
Polyas	37	39	32	38
Split	4	3	4	4
Unmapped	70	63	71	65
Snvs	172	170	170	180
Neos	331	351	325	345

**Fig. 4:**
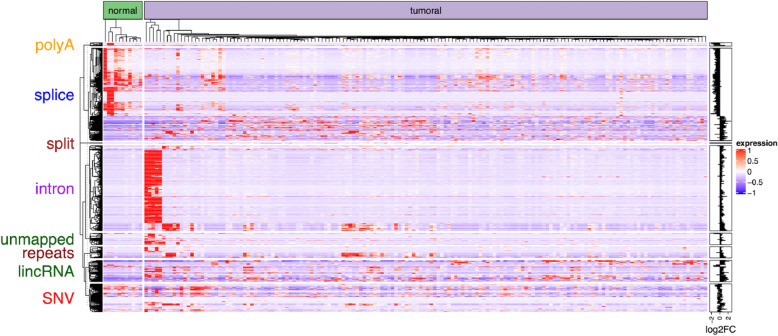
Contig level vs gene level analysis. Category-based heatmap of contigs on normal and tumor tissue samples in ESCA

As shown in Fig. 4, the expression states of those transcriptome events are remarkably different between normal and ESCA tissues. In this case, a major disruption in expression patterns is a result of distinct transcriptional events. Firstly, both low- and high-expression events are included across splicing events in tumor cells. During ESCA, both downregulated and upregulated genes could generate differentially-produced variable shear transcripts. Secondly, significant expression of almost all repeats, lincRNAs, unmapped, introns, and SNVs (Single Nucleotide Variations) is observed in tumor cells but not in normal cells. Such significant expression indicates that a number of untypical transcriptional events take place during the growth of ESCA, and tumor-specific antigens may be contained in those transcriptome events. Lastly, transcriptome events are highly represented in a number of subgroups. This result in subgroups indicates a possible ESCA subtype that can be related to the abnormal regulation of transcription.

### Gene Ontology-based functional analysis over both contigs and genes

**Fig. 5:**
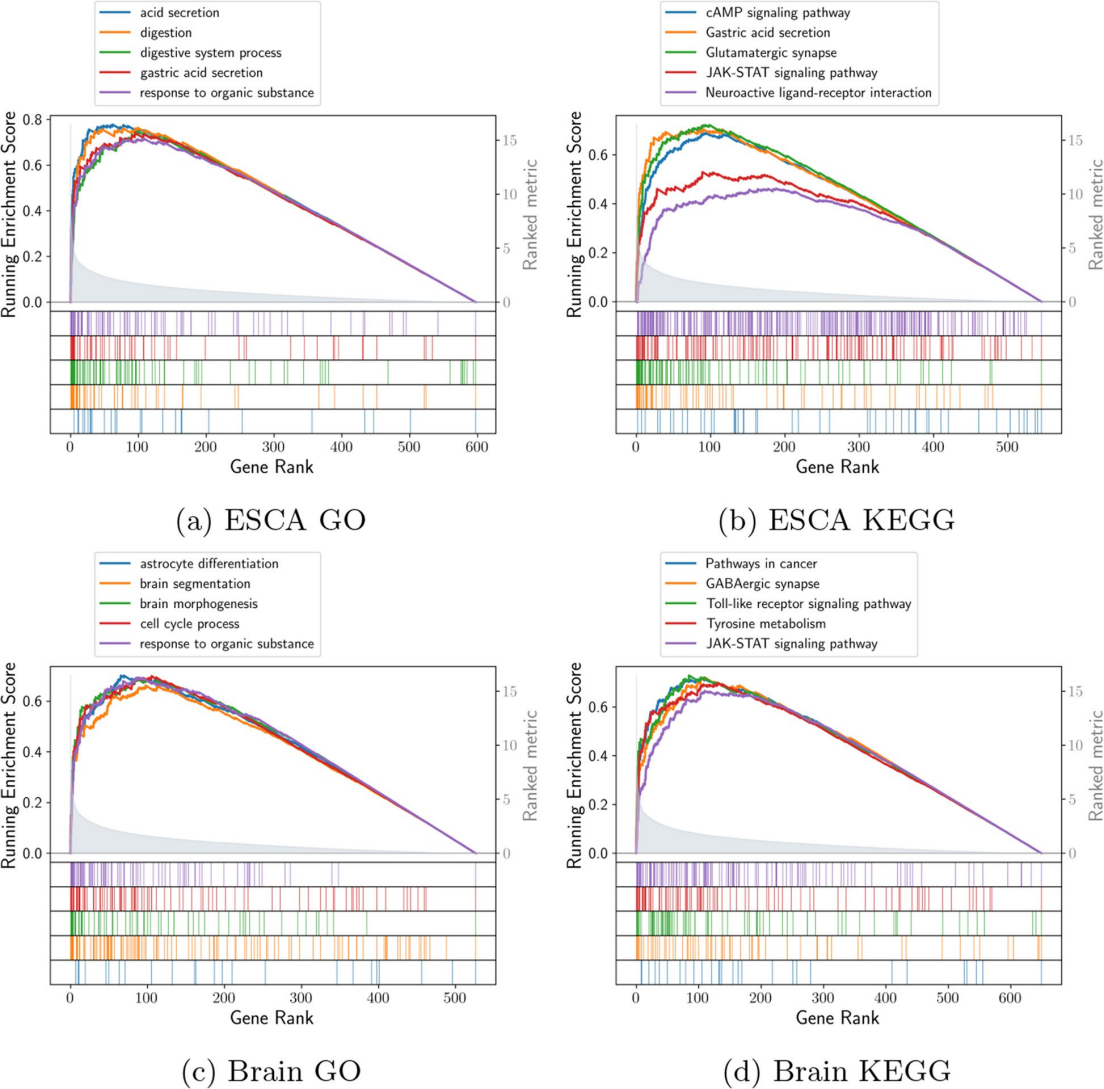
Frequently-upregulated genes functional enrichment in terms of 2 protocols. **a**, **b** logFC (Log fold change) is represented by decreasing order over the x-axis, whereas y-axis shows each function’s enrichment score in terms of Gene Ontology and KEGG pathways respectively for ESCA. **c**, **d** Similar enrichment scores and plots in terms of Gene Ontology and KEGG pathways respectively for glioblastoma

We identified differentially-expressed genes to be suitable with both approaches which was the result of comparison between contig level and gene level datasets. Simultaneously, different utilization connection between transcripts and genes have been discovered. We carried out a Gene Ontology-based functional enrichment over those genes in mastering their biological functions in detail, and Fig. 5a, b present functional enrichment analysis of upregulated differentially-expressed genes in terms of Gene Ontology and KEGG pathways respectively for ESCA and glioblastoma respectively.

Figure 5a shows Gene Ontology-based enrichment findings for 634 DEGs (Differentially Expressed Genes) by comparing their differential usage with the linked transcripts in ESCA tumors. The upregulated genes are mainly associated with digestive system-associated operations and gastric acid secretion. KEGG pathway enrichment [49] is shown in Fig. 5b. According to the results, the upregulated genes are significantly enriched in JAK-STAT, gastric acid secretion, and the remaining cancer pathways [50]. In this case, JAK-STAT and Glutamatergic synapse pathways are previously verified cancer pathways [51, 52], where JAK-STAT signalling is a cornerstone to cancer progression, either as a tumour intrinsic driver of cancer growth/metastasis, or as a modulator of immune surveillance. Similarly, Fig. 5c shows Gene Ontology-based enrichment findings for 695 DEGs by comparing their differential usage with the linked transcripts in glioblastoma. The upregulated genes in glioblastoma are mainly associated with central nervous sytem development and cell cycle. KEGG pathway enrichment is shown in Fig. 5d. According to the results, the upregulated genes are significantly enriched in JAK-STAT, GABAergic, tyrosine, and other common cancer pathways [53–55].

**Fig. 6:**
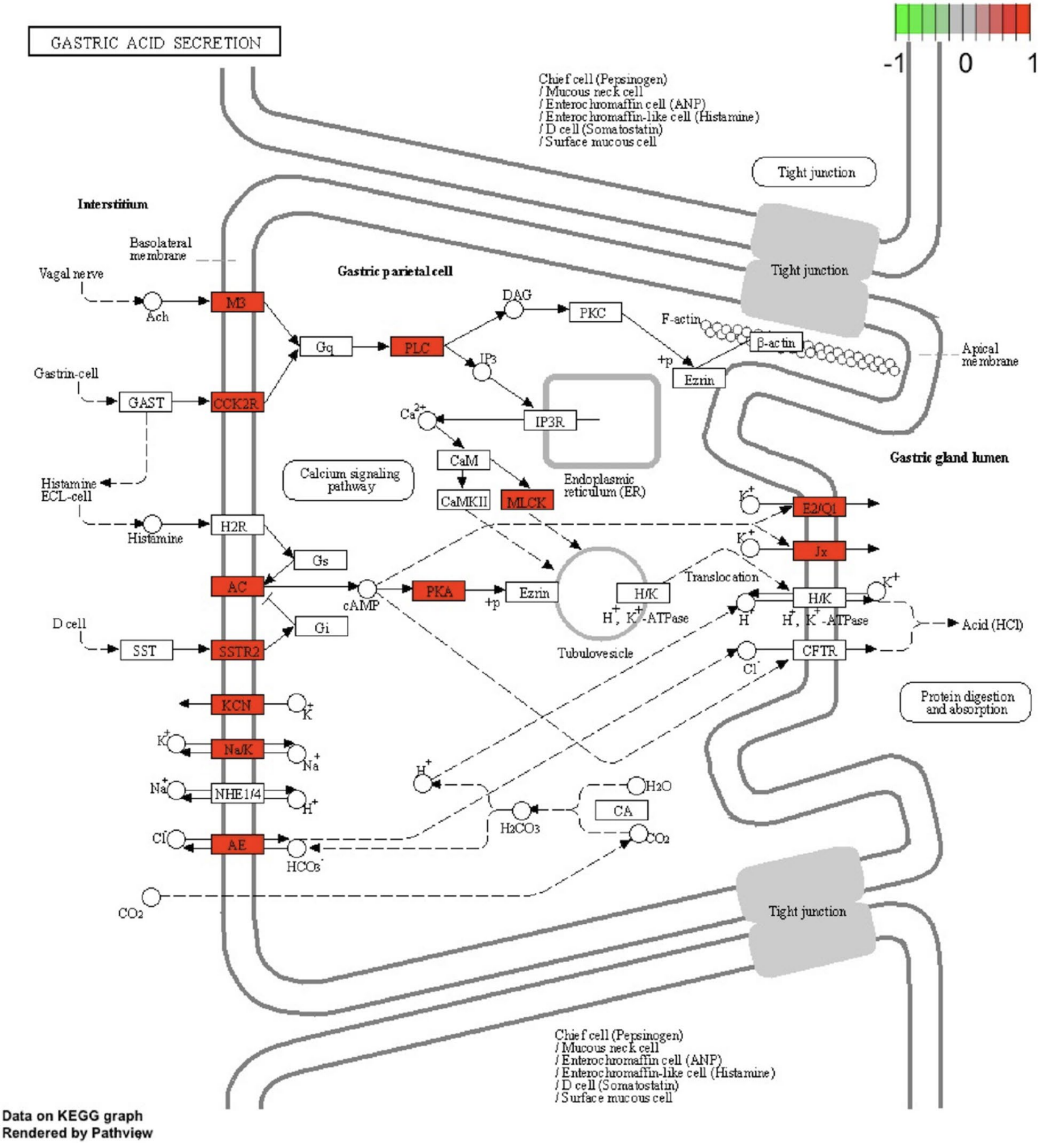
Frequently-upregulated genes functional enrichment in terms of 2 protocols. Sketch of KEGG pathway [49] where red nodes show the upregulated genes in ESCA

Figure 6 plots the distribution of genes and the expression states that are enriched in stomach acid secretion pathways. In ESCA samples, remarkable overexpression of more or less all regulatory genes occur which activates the stomach acid secretion pathway. In this case, major secretion of stomach acid generates a local acidic environment as a significant inflammatory inducer in ESCA samples. Moreover, extra biological pathways are significantly connected to cancer on digestive systems. Some of these extra biological pathway examples are smooth vascular muscle contraction, calcium signaling, insulin secretion, and differentiated cancer pathway. There is a significant relationship between the remaining 3 pathways and cardiovascular illnesses and cardiovascular issues, also inclusive of obesity and diabetes which is connected with an expanded esophageal cancer risk [56, 57].

**Fig. 7:**
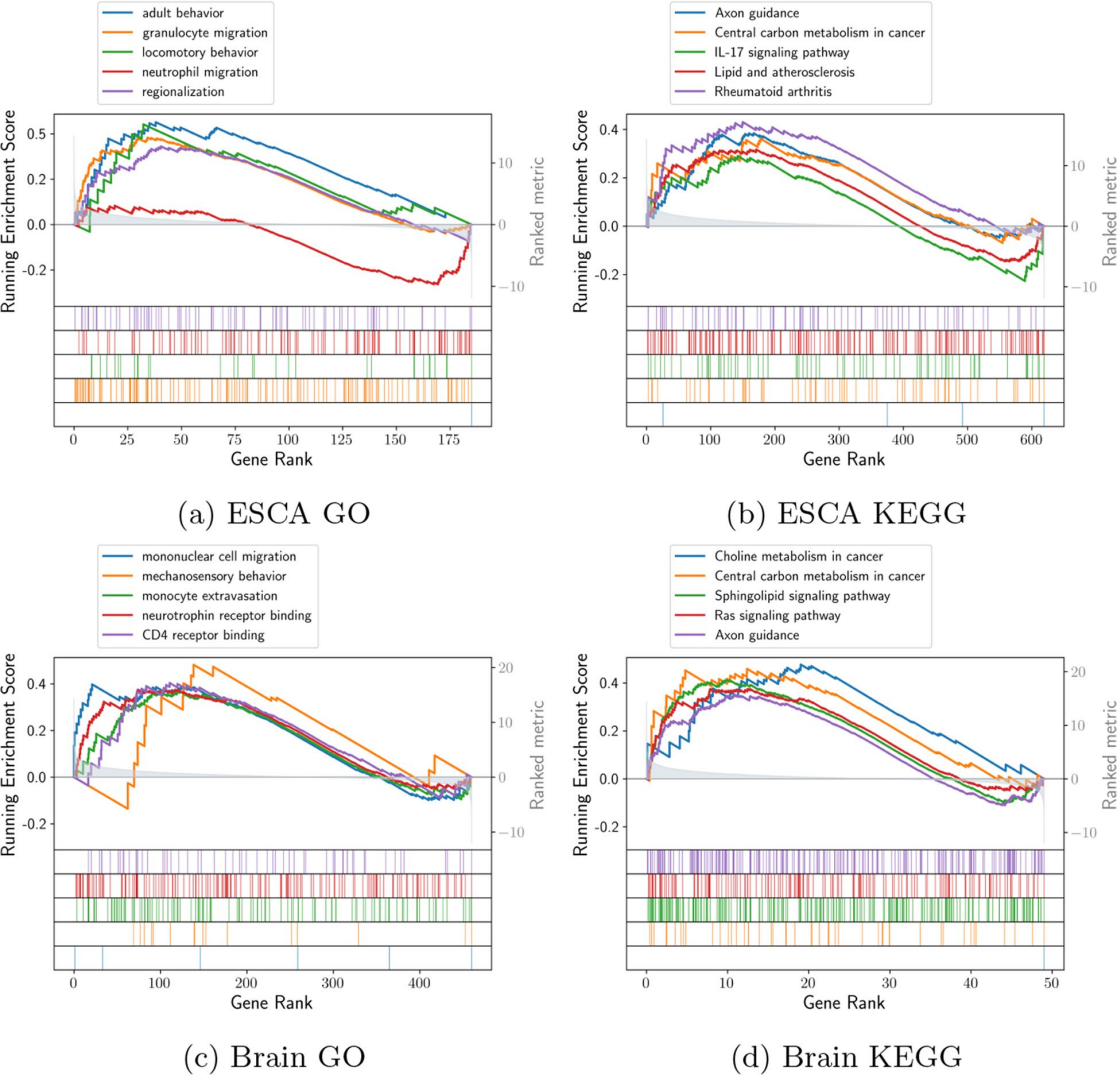
Downregulated genes enrichment in terms of biological functions. **a**, **b** logFC (Log fold change) is represented by decreasing order over the x-axis, whereas y-axis shows each function’s enrichment score in terms of Gene Ontology and KEGG pathways respectively for ESCA. **c**, **d** Similar enrichment scores and plots in terms of Gene Ontology and KEGG pathways respectively for glioblastoma

According to Fig. 7a-d, upregulated genes exhibit inherently contrasting activities than the downregulated genes. Figure 7a shows Gene Ontology-based enrichment results for downregulated genes. The vital obstructed biological functions can be considered as neutral migration and granulocyte migration. Figure 7b plots enrichment results over KEGG pathways for downregulated genes, where interleukin 17 (IL17) signaling pathway, rheumatoid arthritis, lipid and atherosclerosis, and central carbon metabolism in cancer can be seen as examples of supressed pathways [58–60]. In this case, the last 2 pathways are previously verified cancer pathways. The roles of immunology and inflammation can be critical during the prognosis of ESCA, according to the first 3 pathways [61, 62]. Similarly, Fig. 7c shows Gene Ontology-based enrichment findings for downregulated genes in glioblastoma. The vital obstructed biological functions can be considered as neurotrophin receptor binding and monocyte extravasation. KEGG pathway enrichment for glioblastoma is shown in Fig. 7d. According to the results, the downregulated genes are significantly enriched in Choline metabolism in cancer, axon guidance, Sphingolipid signaling pathway, etc [63–65].

**Fig. 8:**
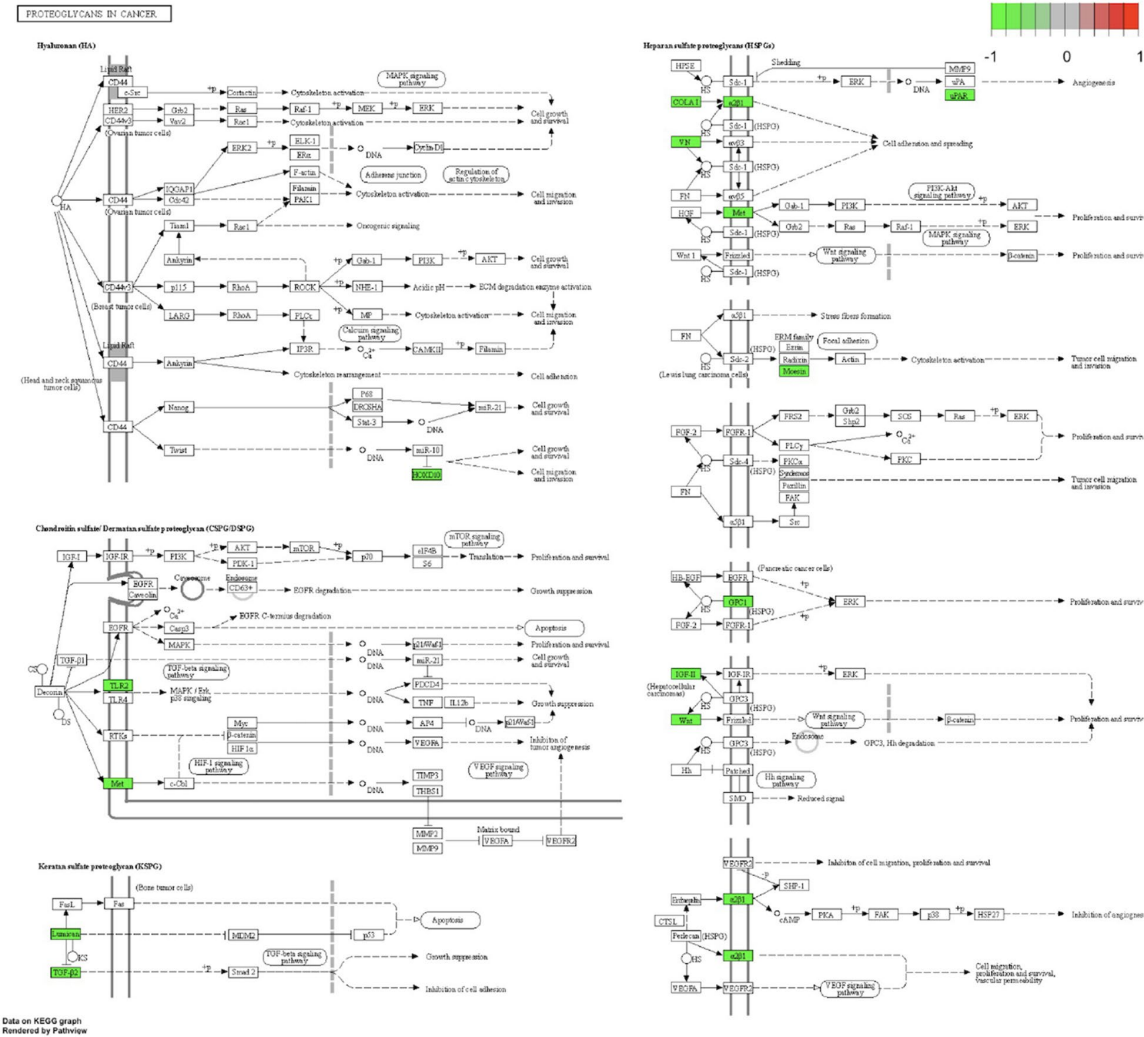
Frequently-downregulated genes functional enrichment. Sketch of KEGG pathway [49] where green nodes show the downregulated genes in ESCA

As in Fig. 8, almost all genes that are enriched in the proteoglycans over the cancer pathway are greatly downregulated in ESCA samples. In line with [66], proteoglycans have an important role in the tumorigenic attributes of esophageal squamous cell carcinoma. The remaining KEGG pathways that are enriched are PI3K-Akt signaling pathway, focal adhesion, and ECM receiver interaction.

### Differential usage analysis

**Fig. 9:**
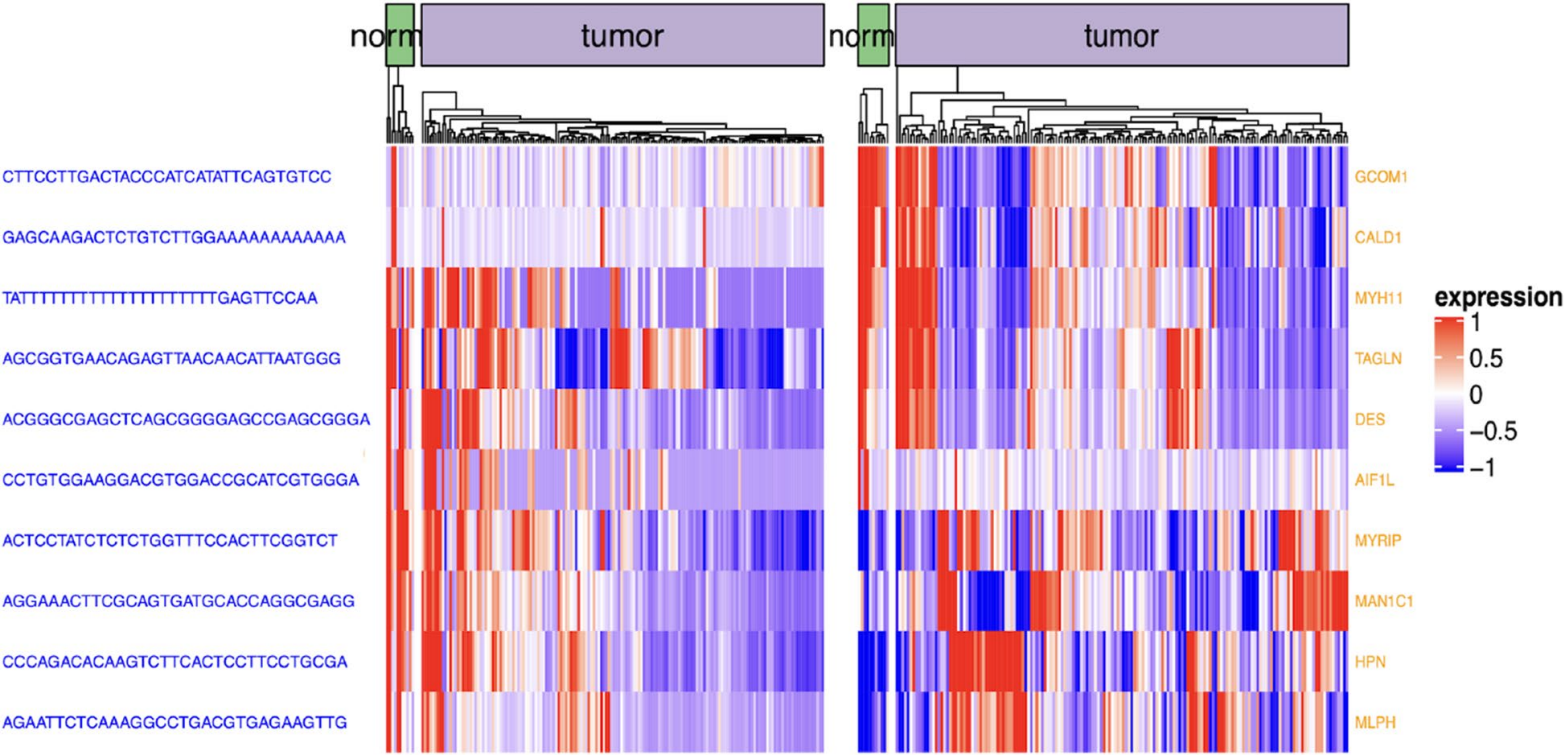
DU events and analysis of the host genes. Heatmap for the topmost ten gene-contig pairs between 2 groups in ESCA

**Fig. 10:**
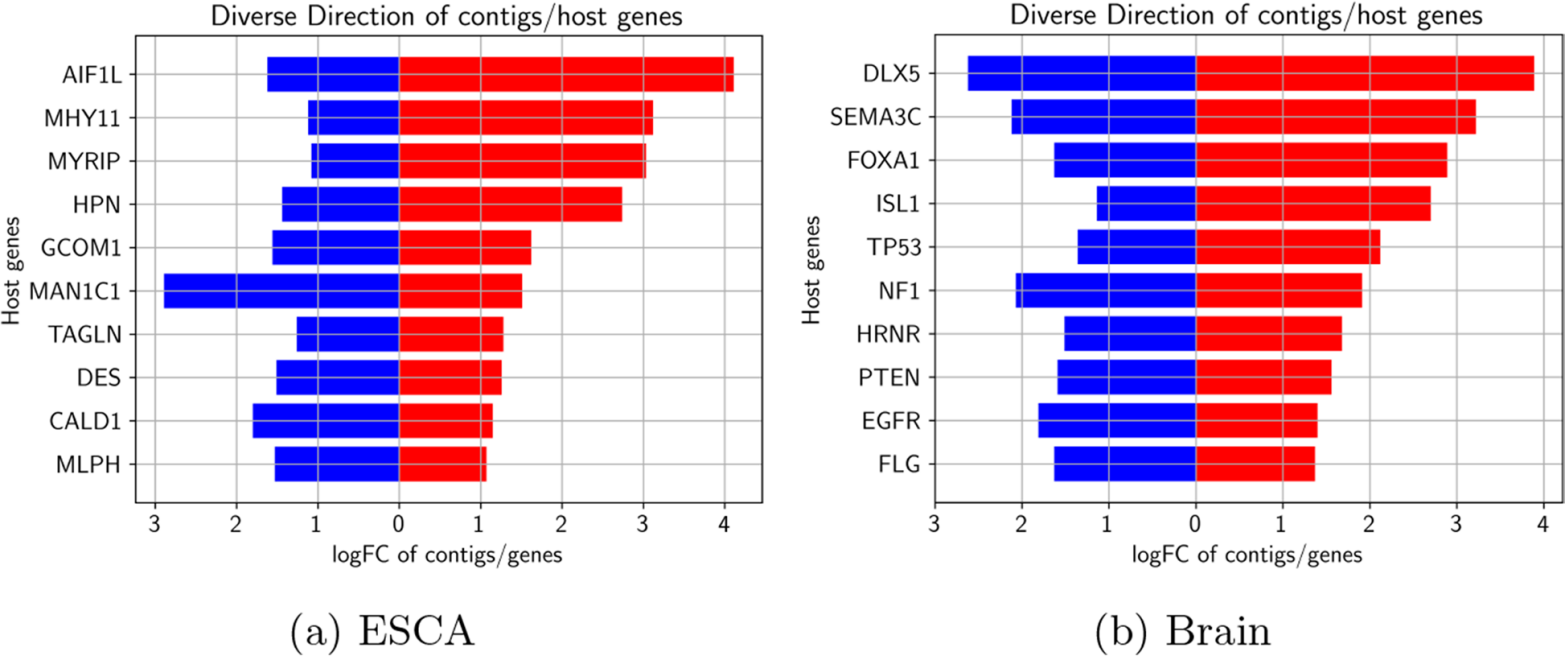
DU events and analysis of the host genes logFC values comparison for every gene-contig pair for both ESCA and glioblastoma

**Fig. 11:**
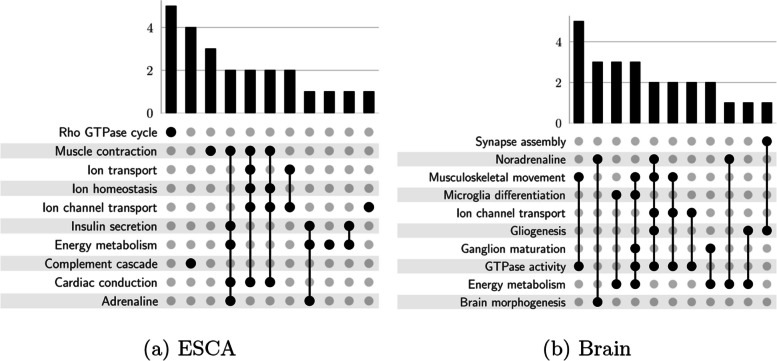
DU events and analysis of the host genes. Enriched biological functions upset plot for both ESCA and glioblastoma

On top of persistent differentially-expressed genes of both protocols, the discovery of multiple specified transcription events of only contigs protocol also takes place. The host gene of those transcriptional events has either the reverse expression direction as contigs or not differently-expressed at all. Disease-associated regulatory anomalies during transcription frequently go along with that DU event. So, we have thoroughly analyzed the whole set of contig protocol-specific DU cases. As seen in Fig. 3a, we have identified 148565 downregulated contigs and 191423 upregulated contigs where 7487 non-upregulated and 5352 non-downregulated contigs belong to hosts. Additionally, we have identified 8124 and 76234 upregulated and downregulated pairs of contigs and host genes respectively which exhibit a similar regulatory movement tendency. As seen in Figs. 9, 10 and 11b, we use Wilcoxon test [48] to calculate the expression difference between contigs and matching host genes for every DU contig-gene pair. Afterwards, screening the ten most statistically significant contig-gene pairs took place.

As seen in Fig. 9, contig sequences exist on the left side, the corresponding host genes exist on the right side, and the gene expression heatmap of contigs and gene expression levels exist in the middle of the plot. The expression movement tendency of the main transcripts is expressed via the heatmap at a gene level. Without any surprise, the contigs attributed to DU events change remarkably from their corresponding host genes. Figure 10a, b show the logFC (Log fold change) values that correspond to gene levels in blue color, wheeras at a contig level, logFC is represented by red color for ESCA and glioblastoma respectively. For both ESCA and glioblastoma, these identified differential genes are previously known to be affective in cancer formation and prognosis [67, 68]. As seen in Fig. 11a, b, once we select the genes that correspond to the top 100 most significant contigs in accordance with DU p-values for biological functional enrichment, then we have additionally clarified these events biological activities. We demonstrate that DU events are largely part of the immune response and insulin secretion for ESCA tumors. On the other hand, DU events are largely part of the brain morphogenesis and energy metabolism for glioblastoma.

### Inferring new RNA events as prognosis measures

**Fig. 12:**
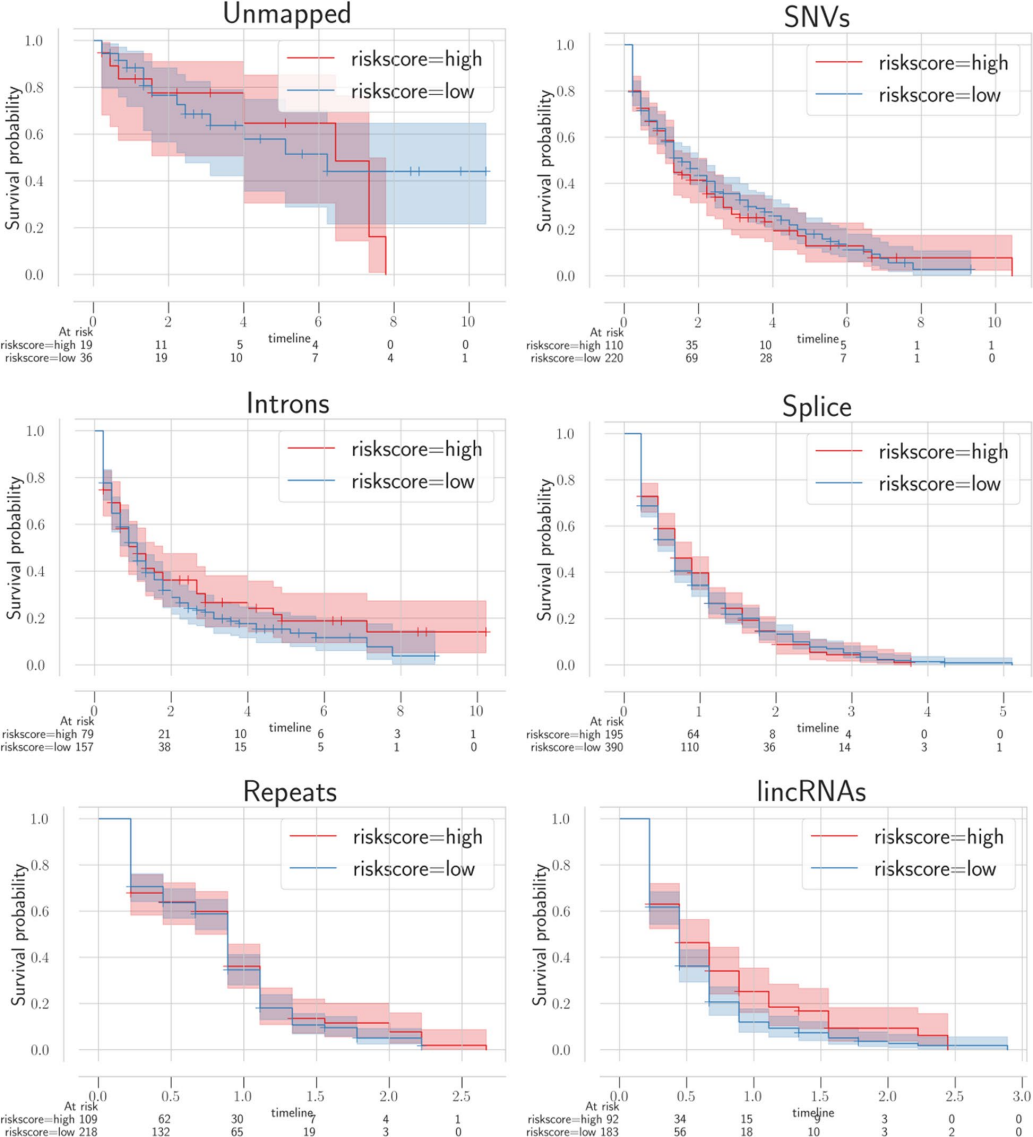
Survival analysis of multiple new events in ESCA where every panel matches with a single type of variant event. The red and blue curves define the patients survival results with varying risks

**Fig. 13:**
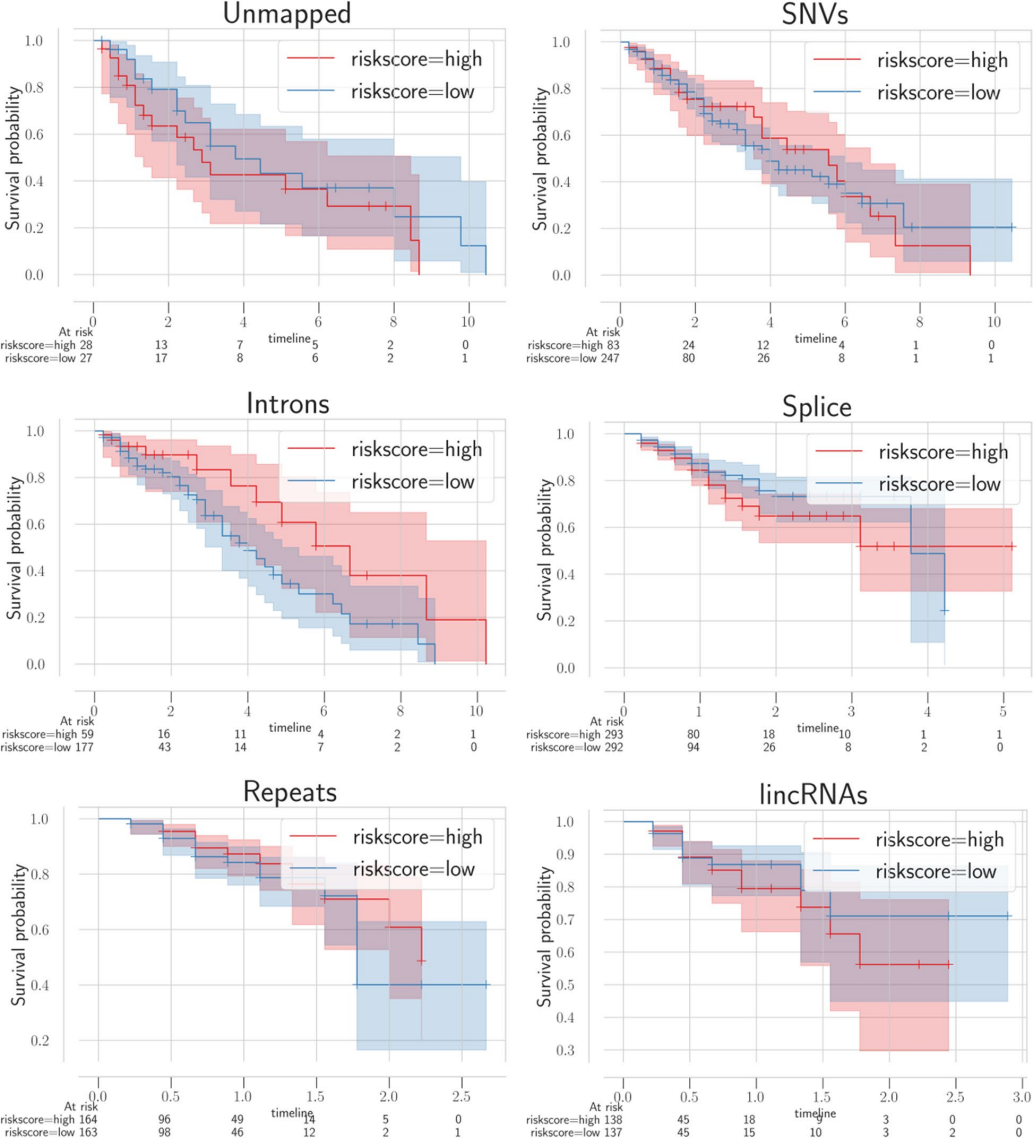
Survival analysis of multiple new events in glioblastoma where every panel matches with a single type of variant event. The red and blue curves define the patients survival results with varying risks

We have identified plenty of ESCA-associated transcription events that include lincRNA, intron, split, SNV, among others by using mapping-free and alignment-free approaches. We can use comparison tools to map the happenings of these events, and research has identified alternative splicing, SNV, and the remaining factors connected to the prognosis of ESCA. Nonetheless, as seen in Table 1, only a limited number of studies analyzed the connection between great many transcriptome events and the prognosis of ESCA simultaneously. Those transcription events associated with ESCA are treated as novel events since these events are previously unannotated. We run univariate Cox regression of various novel RNA event types in our study. Afterwards, we carried out a multivariate Cox regression analysis over the related events extracted by univariate Cox regression, and applied Kaplan-Meier (KM) curves to bring out survival time variations among different risk samples as seen in Figs. 12 and 13 for ESCA and glioblastoma respectively.

**Fig. 14:**
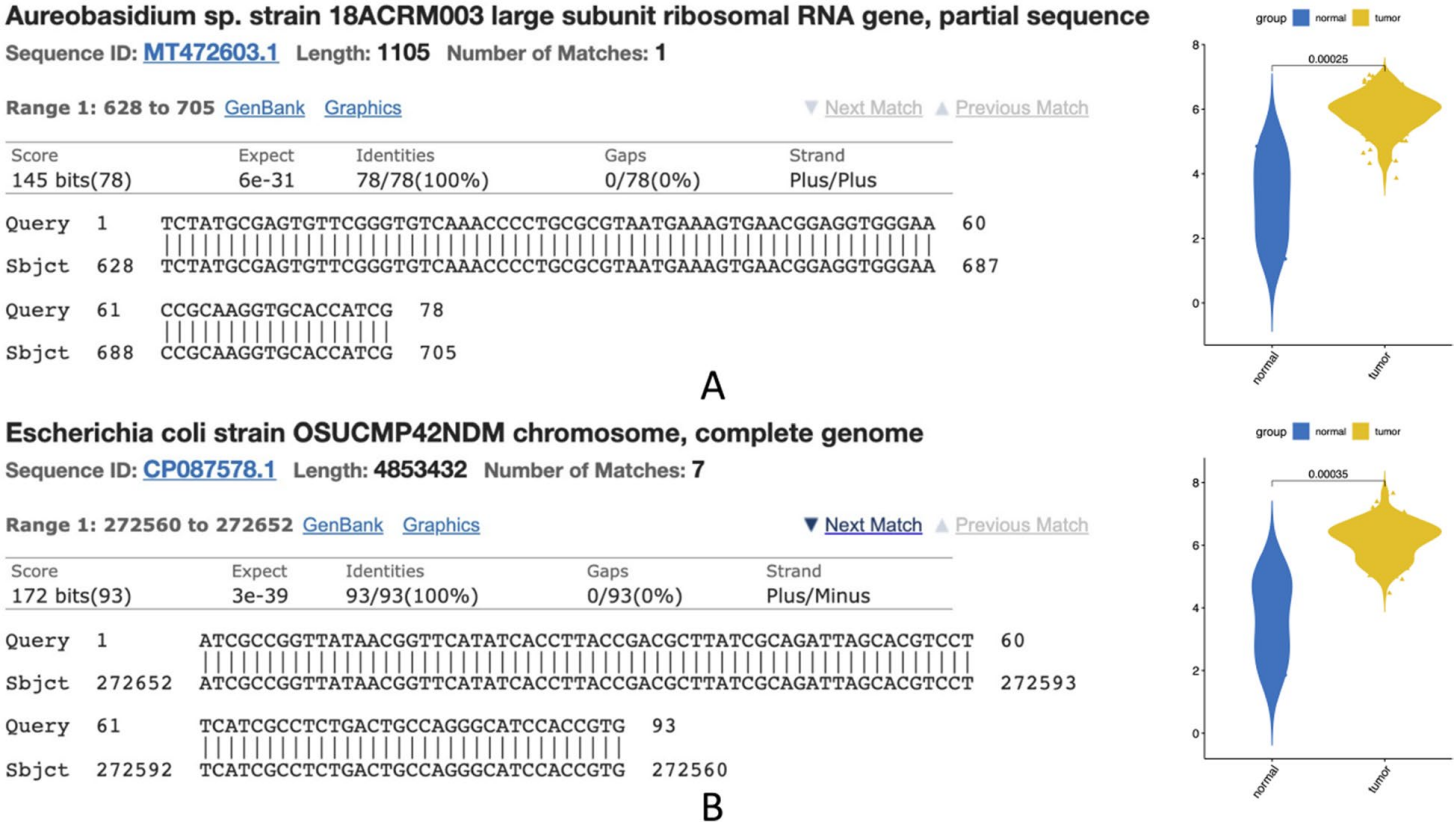
Unmapped transcripts in ESCA that are aligned to bacteria via BLAST and the expression values of 2 representative contigs

In KM curves, there exists a horizontal axis for survival duration in terms of year and a vertical axis for the probability of survival. We color low-risk and high-risk samples with blue and red colors respectively. Here, we can see that different transcriptional event types that are detected by using an alignment-free approach have a high correlation in terms of prognosis. Particularly, we have identified unmapped transcripts related to the patients final survival. We examined those unmapped transcripts in detail by utilizing alignment tools. For the first alignment-based annotations, we used a splice-aware aligner GSNAP. A different BLAST aligner is used for screening the unmapped events in detail. In terms of 72 unmapped events extracted by GSNAP, we could align 18 out of 70 events to the human genome via BLAST without considering the mismatches. 43 contigs are found to be connected to bacteria living in the digestive system. Lastly, the remaining 11 contigs do not have any known organism in the traditional databases. Figure 14A, B represent 2 unmapped contigs, that are upregulated compared to the normal tissues in ESCA samples. This upregulation proposes microbial infection as an important factor in esophageal cancer’s start and prognosis. On the contrary, conventional alignment-based methods mostly ignore sequences which could not be aligned to a reference genome.

### Neoantigen candidates

**Fig. 15:**
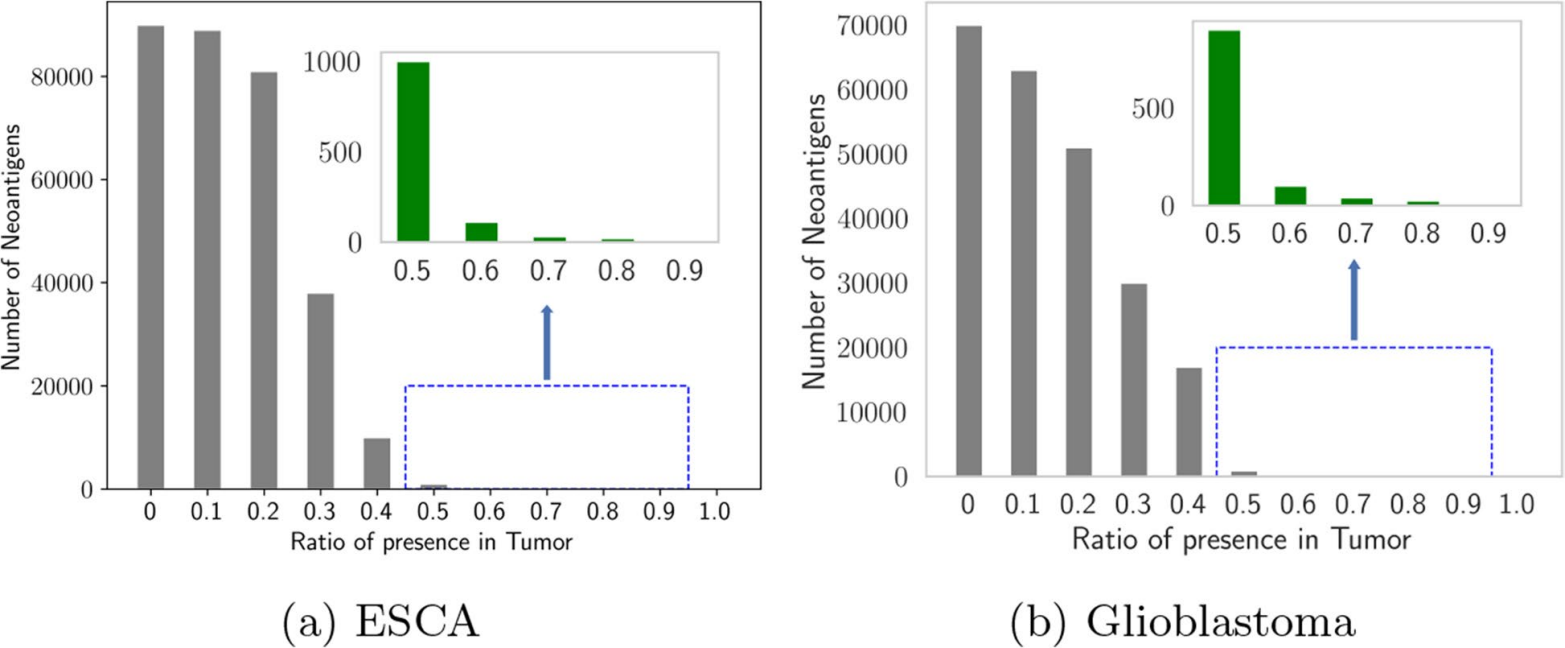
Estimation of neoantigens. The number of prospective neoantigens occurring in the patient population with different percentages in both ESCA and glioblastoma

**Fig. 16:**
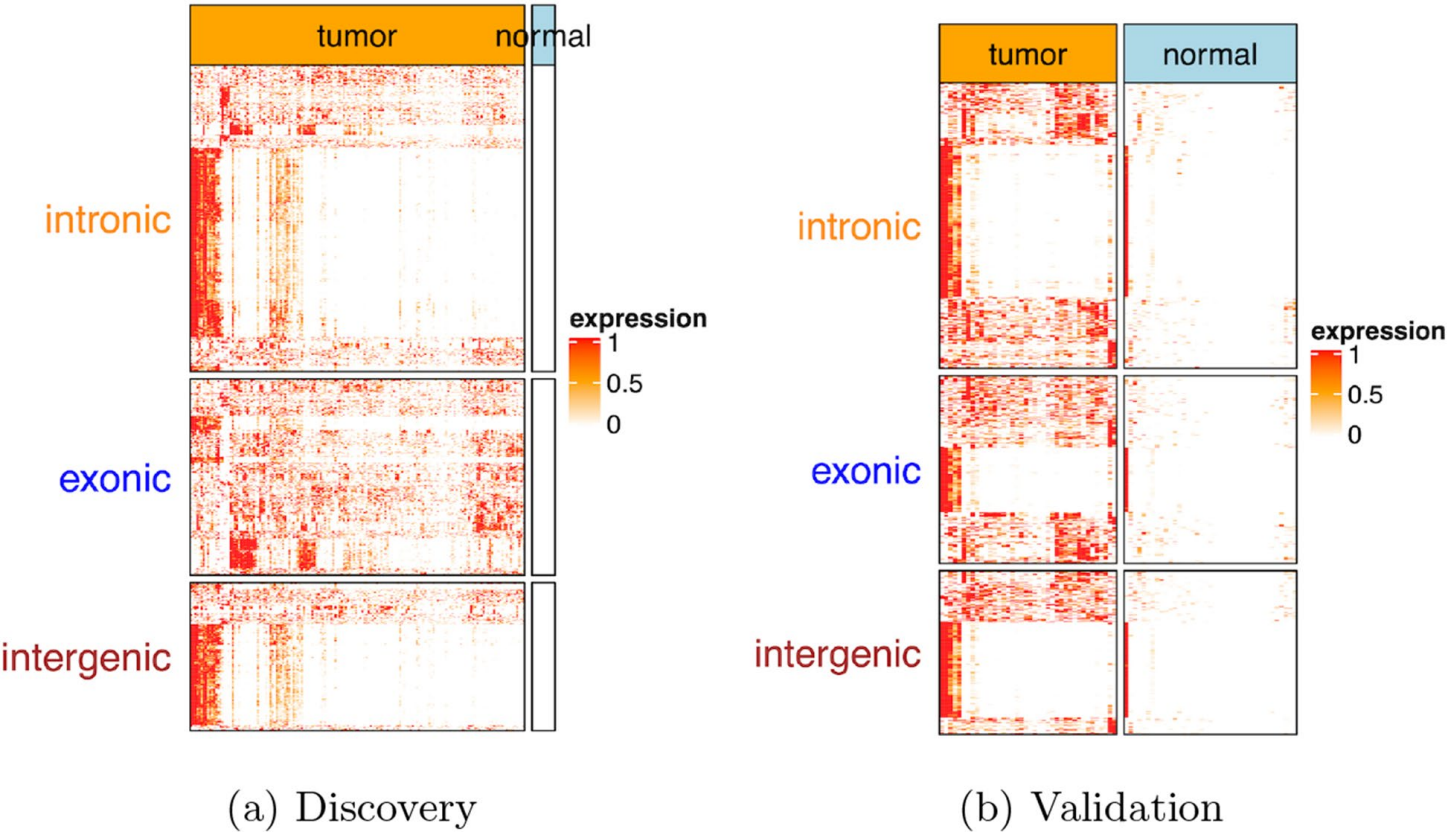
Estimation of neoantigens. Expression of chosen neoantigens between cancer and normal tissues in both validation and discovery cohorts for ESCA

We have assessed sequences whose expressions are specific to tumor tissues instead of normal tissues for all transcription-based events. As seen in Fig. 15, we have inferred a considerable number of neoantigens in a very small fraction of tumor tissues for both ESCA and glioblastoma. Recurring neoantigens extracted in patients with tumors are considered to be clinically quite useful [69]. We apply a robust criterion to identify the most commonly occurring neoantigens over malignancies: Firstly, neoantigens should exist in at least $50\%$ of all tumor tissues when they lack in normal tissues. Secondly, we selected candidates over 1216 neoantigens. The primary origins of those contig sequences are intronic, exonic, and intergenic regions. As seen in Fig. 16a, b, we have verified the expression of all recurring neoantigens over both ESCA and validation datasets.

Figure 15 outlines the frequency of neoantigens which is associated with the recurrence ratio in cancer tissues, from tiny to large for both ESCA and glioblastoma. According to the green barplot, more than half of the cancer tissues have neoantigens across both cancer types. The safety of those neoantigens has been established as well by verifying the neoantigens expression across both ESCA and glioblastoma discovery and independent validation datasets. In this case, the chosen neoantigens should not be expressed for any normal noncancerous tissue over the ESCA and glioblastoma discovery cohort. Even though a number of neoantigens are still expressed silently in normal tissues, these antigens manifest a significantly lower expression tendency than malignant tumor tissues in the independent dataset.

**Fig. 17:**
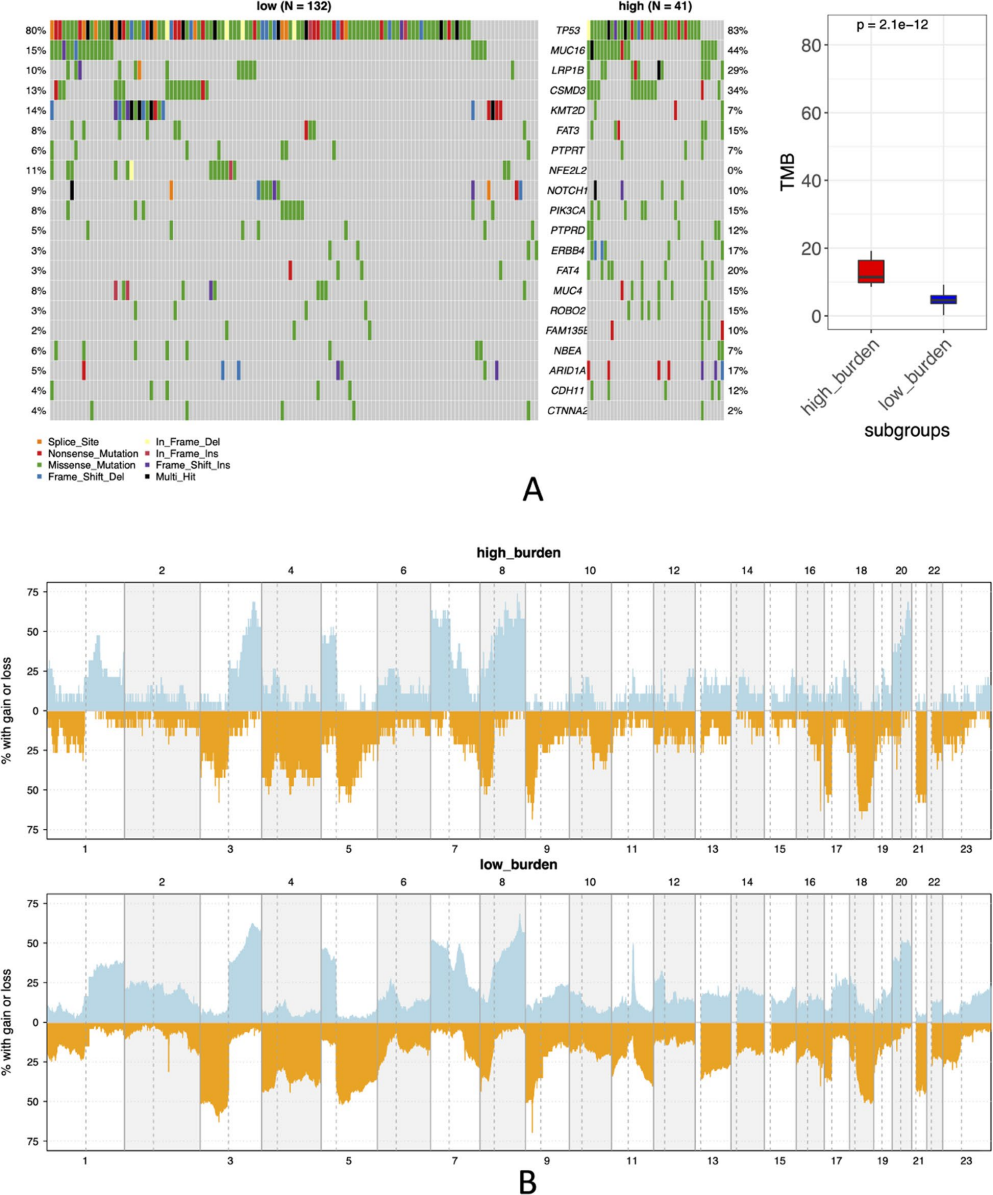
Copy number variation (CNV) and burden of mutations across 2 subgroups. **A** Mutational burden and the distribution of mutations among high-burden and low-burden subgroups. **B** CNVs throughout 24 chromosomes between 2 subgroups where orange and blue indicate deletion and duplication respectively

Meantime, an important discovery was that a number of ESCA and glioblastoma patients exhibit higher quantities of antigens that are specific to tumors than the remaining ones. In line with these results, immunological subgroups might exist which respond quite well to the anti-tumor vaccines in patients with both cancer types at the immune response level. Therefore, ESCA patients are partitioned into 2 subgroups depending on antigens specific to tumors. Then, as seen in Fig. 17, we assessed the burden of tumor mutations and instability of the genome over those 2 groups.

As can be anticipated, remarkable differences in CNVs and mutations exist among the 2 sample groups having different immunological subtypes. Subgroups that expressed extra tumor-specific antigens incorporate a greater number of CNVs and mutations. So, immunotherapy may be a better option for patients within that category [70, 71].

## Discussion

Here, we have focused on determining a great number of exogenous pathogenic microorganism sequences, esophageal cancer-associated variants, and new transcripts without an annotation via a reference-free and alignment-free procedure of ESCA and glioblastoma single-cell RNA-seq dataset. Those transcription-associated events exist as part of the whole genome, which include intergenic regions, coding regions, non-coding regions, etc. Conventional gene level transcriptome procedures could evaluate the biological processes associated with phenotypes from the gene level, without seeing the more detailed transcript level. On the other hand, traditional transcript level procedures count on proper alignment of reads to the genome, so these procedures may solely concentrate on the coding region’s variations without identifying the transcripts that are not annotated. Even though recent research progress has focused on overcoming the reference sequence’s limits via de novo transcriptome assembly, such a de novo assembly procedure will cause a great many assembly errors as well. Additionally, the assembly procedure also requires aligning sequences to the assembled reference sequence afterward.

To our best knowledge, none of the existing studies has conducted a detailed analysis of the single-cell transcriptome datasets of esophageal and glioblastoma cancer tissues, and the existing studies have not assessed all transcripts over the whole genome in a systematic way. Consequently, our proposed analysis has great potential since we can gather different types of transcriptome events independently without taking their origins into account. In this case, by using a mapping-free analysis technique, we can discover the origins of new epitopes more properly. The identified tumor-specific antigens are especially expressed across several ESCA and glioblastoma patients and they have been reproduced in independent validations datasets. Our reference-free and alignment-free analysis has multiple different advantages. Firstly, the whole matrix dataset might be expressed as a single matrix dataset to efficiently carry out matrix operations, rather than following a more traditional approach that run every sample independently. So, we can better optimize for time and computational resources. Secondly, reference-free and alignment-free approaches might be especially charming in metatranscriptomics, where RNAs are collected in an environment with unknown archaebacteria, bacteria, or eukaryotic species. Lastly, our procedure ensures that any RNA that exists particularly in a sample subset will be caught without considering its origins.

There are limitations to this current research. Firstly, plausible errors exist as part of assembling differentially-expressed k-mers into contigs. Nonetheless, our k-mers length is 31 bp so the longest contig for a mutation position after assembly is 61 bp. In this case, 61 bp is still greatly less than the full-length transcriptome. As a result, this study’s assembly procedure has a lower error probability compared with the de novo assembly. Even though transcripts could be evaluated at a more detailed level, we use Kallisto to evaluate the gene level’s quantification. Kallisto, as a reference-free approach, does not count on reference sequences so its accuracy is lower than the traditional quantification approaches. But, such performance decrement does not significantly affect our conclusions since our study’s results do not mainly depend on the gene level.

## Conclusions

Traditional procedures that rely on aligning sequences to a reference have a number of limitations. However, most of the tumor formation and prognosis dynamics could not be demonstrated via alignment-based procedure results. The alignment-free and reference-free methods are more effective and broad variant callers, even if they cannot fully replace the traditional methods with regard to accuracy. Those alignment-free approaches have many advantages in terms of the discovery of novel variants and complex genomic elements such as repeats. As a result, in the future, combining more conventional alignment-based and alignment-free procedures will have comprehensive promises to reveal tumor dynamics.

## Data Availability

The single-cell RNA-seq discovery datasets for ESCA are obtained from Gene Expression Omnibus (GEO) with accession number GSE160269 and Sequence Read Archive with accession number SRP327447. For ESCA, the validation datasets with accession PRJNA374673 are obtained from SRA database. Similarly, single-cell RNA-seq discovery datasets for glioblastoma are obtained from European Genome-Phenome Archive with accession number EGAS00001004422. For glioblastoma, the validation datasets with accession PRJNA869596 are obtained from SRA database. Processed datasets and source code of the analysis in this research are available on https://github.com/seferlab/cancerreferencefree.
